# On the Efficacy of Pressure by the Application of Bandages in Cases of Phlebitis, Phlegmonous Erysipelas, Burns, and Wounds in Dissection

**Published:** 1829-12

**Authors:** Alf. Velpeau


### COMPRESSION.

*On the Efficacy of Pressure by the Application of Bandages in Cases of Phlebitis, Phlegmonous Erysipelas, Burns, and Wounds in Dissection.* By ALF. VELPEAU. (Condensed from the Revue Médicale, Juin 1829.)

HOWEVER promptly and energetically we may treat patients who are labouring under either of the above maladies, by any of the ordinary modes of practice, it is very certain that we shall frequently fail. We are led, then, both from humanity and our love of science, to seek for new means of affording relief. The success of the treatment adopted in the following cases will no doubt induce other surgeons to give it an impartial trial.*

CASE I. M. G. was attacked, in July 1826, with severe pain in the left inferior extremity, and with all the ordinary symptoms of inflammatory fever. Twenty-four hours after, the whole of the leg and thigh were greatly swoln, excepting the outside of the latter. The skin was partially red, especially in the course of the internal saphena vein, and on the outside of the leg. The least motion or pressure gave excessive pain. In the leg the vein could not be felt, on account of the swoln and tense state of the integuments; but, at the upper part of the thigh, where the inflammation was less severe, the saphena formed an evident round cord, which was easily detected under the skin. The pulse was full, quick, and strong; skin hot and dry; great thirst; tongue white; no pain in the chest or belly. The only fact that could be ascertained which could bear at all upon the origin of these symptoms, was that the patient, in making a violent exertion in bathing three days before, had felt a cracking and pain in the lower part of the affected leg. Besides this, a slight excoriation on the outside of the heel, which had now entirely healed, had been in a state of suppuration for a week. These were the only circumstances which appeared to have any connexion with the state of the patient.

He was bled to twelve ounces; and, for the purpose of arresting the progress of the inflammation, sixty leeches were applied principally to the bend of the groin. Emollient poultices; warm bath.

On the third day, the pulse was less full, but still as quick; thigh not so much swoln, but the leg more so than

* M. Velpeau has published many other cases, which confirm the efficacy of this practice. Vide Archives Générales, June 1826, p. 192, and July 1826, p. 395.—EDITORS.

before. Forty leeches applied below and around the knee; warm bath repeated.

On the fourth day, the inflammation remained unabated; pain excessive; and the leg, which felt very heavy, now appeared to be the seat of general phlegmonous erysipelas. On the internal part of the thigh there were several red blotches, extending to near the groin; the hardened and enlarged vena saphena could be distinctly felt to within six inches above the knee. The superficial inguinal glands were slightly painful and swoln. General symptoms unchanged.—Twenty leeches to the groin, and the same number to the leg.

Fifth day: no evident abatement of the local symptoms; fever somewhat diminished. M. Marjolin was now consulted. M. Velpeau proposed a compressive bandage, but it was deferred for twenty-four hours, during which time the inflamed parts were covered with compresses wetted with cold water, but without any good effect.

Sixth day, at ten o'clock in the morning, M. V. surrounded the limb, from the toes to near the groin, with a circular bandage moderately tightened, and afterwards wetted it with cold marshmallow water. The pains continued to increase in severity for three or four hours, at the upper part of the foot and internal malleolus, and remained in these parts until the next day; but towards evening every other part was much less painful.

Seventh day.—Patient has slept. Feverish symptoms have disappeared, and perspiration has broken out. The thigh is now neither red nor swollen; the vena saphena can still be felt, and remains hard and slightly painful. In the leg the swelling and other symptoms of erysipelas have diminished more than one half in degree; but, around the internal malleolus, the redness, pain, and tension, were unchanged, and presented every appearance of an approaching collection of matter, but, as no fluctuation could be detected, the bandage was again applied.

Eighth day.—Has not passed so tranquil a night. The back part of the foot and above the ankle are more painful and swoln. In the former situation there was evidently an abscess, which M. Velpeau opened, and gave exit to a small quantity of matter. Fifteen leeches were applied around the ankle, where there was no appearance of fluctuation. But little swelling or pain in the other parts of the leg or thigh. Pressure continued, except upon the part in pain, which was covered with a poultice.

Ninth day.—The local bleeding had produced no relief.

The abscess was nearly closed. The bandage was applied no higher than the knee.

Eleventh day.—M. V. thought he detected pus in the painful part. An incision was made nearly to the tendon of Achilles before the abscess was opened, and about an ounce of well-formed pus escaped.

From this time the cure proceeded uninterruptedly, and the patient was enabled to resume his accustomed occupations. The vena saphena major, however, remained hard for a considerable time, and the limb but slowly recovered its natural strength; and, although a laced stocking was worn, it still remains occasionally painful and slightly swollen, especially after any fatigue, or when the general health is deranged. This may probably depend upon the total or partial obliteration of the principal superficial vein of the part.

Case II. A strong and well-formed labourer wounded himself, in the course of the cephalic vein of the thumb, with the point of a vine-knife, in October 1826. The wound, which was very small, suppurated without giving rise to much pain, until the 10th November. The parts around it then became affected with severe inflammation, which extended in tortuous streaks over the back of the hand.

On the 15th, M. Nivert saw the patient. The whole of the metacarpal plexus of veins was of a deep red colour: the intervals between these highly coloured cords were equally inflamed, but they presented only a slight erysipelatous redness. The swelling was not considerable, although the pain was acute, and there was some fever.—Twenty leeches, and afterwards a bread poultice, were applied to the hand.

16th.—The veins of the forearm are now implicated, and form red and highly sensible cords throughout the whole extent of this part of the limb, the swelling of which is becoming considerable. There are some patches of erysipelatous inflammation on the upper arm, but the pain is there much less severe than below the elbow and on the hand. The pulse is strong, full, and frequent; skin burning and dry; face flushed —Twelve ounces of blood to be taken; poultices continued.

17th.—Pulse 120, not quite so full as yesterday; fever intense; patient confused in his ideas, and agitated. The swelling has extended to the axilla; the glands are somewhat enlarged and painful. In fact, the whole limb is now affected with very severe phlegmonous erysipelas, the inflammation being most intense throughout the course of the superficial veins. M. Nivert, who had witnessed many of the cases previously related by M. Velpeau,* determined to make trial of pressure. He forthwith surrounded the whole arm with compresses wetted with marshmallow water, having first applied a gauntlet,† and filled the palm of the hand with lint. He then, with a long bandage, about three fingers broad, made an exact, regular, and moderate pressure over the hand and arm, up to the shoulder. The patient was much alarmed at the application of a bandage upon parts so exquisitely painful, and he complained severely for three hours; but towards evening his fears were in a great measure relieved, and the feverish symptoms had much subsided. The bandage was again wetted, in order to increase the compression without removing it.

18th.—Has had a good night. Pulse eighty; slight perspiration. Patient is cheerful, and suffers but little. The swelling and redness of the limb are greatly diminished. The axilla and the upper part of the arm especially are nearly of a natural appearance, and the basilic and cephalic veins can now be felt like hard and knotty cords. The bandage was again applied so as to press firmly upon the hand, and gradually less as it was carried upwards towards the shoulder.

20th.—Great improvement. But little inflammation above the elbow; swelling of the forearm has nearly disappeared, but the back of the hand is still painful, and pits on pressure. Wound of the thumb has healed.—The gauntlet no longer to be applied. Graduated compresses to be placed on the back of the metacarpus, to secure a proper pressure from the bandage, which was now carried no higher than the elbow.

In three or four days this patient was completely cured.

Case III.—M. Caoly, a medical student, three days after having pricked himself on the middle finger of the left hand in dissecting, felt a numbness and uneasiness over the whole of the arm. The next day the injured finger was red, painful, and swollen, and the same appearances were extended to the hand, upon which twenty-five leeches were applied.

M. Velpeau saw him on the third day. All the veins of the metacarpus were of a deep red colour, and those of the

* Archives Gen. Juillet 1826.

† *Gantelet.* A kind of bandage which covers the hand and fingers like a glove, from whence the name.—Ed.

forearm, although less hard and painful, were highly inflamed. The inflammation had now extended beyond the elbow; there was severe fever, pulse 100; the whole of the cellular tissue of the finger, hand, and wrist, was affected by the inflammation. M. V. proposed compression, which was submitted to with some repugnance. Although the middle finger alone was greatly swollen, the gauntlet was employed, and a roller was then applied as neatly and methodically as possible, from the finger to the shoulder. No exacerbation of the symptoms followed. In the evening, the pain, heat, and fever, had much diminished. The bandage was wetted with brandy and water, to tighten without removing it.

Fourth day.—No fever. The patient called upon M. Velpeau to be dressed. The erysipelas had disappeared from the arm, and nearly from the forearm, but still the superficial veins of the latter part appeared like red streaks, and were slightly painful. The hand still remained swoln.

The same treatment was continued the fifth and sixth day. The seventh, there remained only a slight swelling of the finger originally injured. On the eighth day the cure was complete.

Case IV.—M. F., a medical student, had had a slight excoriation on the thumb for some days, when he consulted M. Velpeau. The thumb, hand, fore and upper arm, were swoln and painful; streaks of a livid red colour marked the course of the cephalic and other veins around the thumb; but these inflamed lines were less prominent than in the three preceding cases, although the glands in the axilla were as sensible to the touch, and as perceptibly enlarged. It was presumed that this was a case of inflamed lymphatics rather than of phlebitis. The bandage was, however, applied as before mentioned, from the fingers to the shoulder; and it was wetted in the evening with spirituous lotion. As soon as it was applied, the pain began to diminish.

On the following day, the axillary glands could scarcely be felt; the swelling of the arm had disappeared, and there was neither redness nor pain of the forearm or hand. The bandage was continued until the fifth day, when the cure was perfect.

Case V.—In February 1828, M. O., a medical candidate, about thirty years of age, who had habitually enjoyed good health, excoriated the thumb of the left hand in placing a putrid body upon a table, for M. Velpeau's lecture on operative surgery. On the first day the wounded part was slightly painful; but not until the morning of the third day did any symptoms arise which attracted much attention, when a rather severe shivering occurred, and at noon the face was pale, the features drawn, and the eyes dull. M. O. felt himself very uneasy, and retired to bed. The thumb had now begun to swell, and there was a stiffness of the arm. A fainting fit, followed by high fever and a very restless night, succeeded.

On the morning of the fourth day, M. Velpeau saw this gentleman. His pulse was 115, strong and hard; skin dry and burning; tongue tumid and white; chest and abdomen free from pain; the face was slightly tinged with a dirty yellowish colour; the hand and all the fingers were livid and much swoln, and the forearm and arm were also attacked, but in a less severe degree. The skin was highly coloured up to the elbow, but neither the veins nor lymphatic vessels could be felt. The pain was most severe upon the thumb and back of the hand, near the root of the middle finger. Twelve ounces of blood were taken from the arm, and emollient poultices applied.

In the evening the general symptoms were the same: the axillary glands were now inflamed, and the erysipelas had extended to the shoulder. The hand and wrist were so much swoln and livid as to threaten to become gangrenous. M. Velpeau applied first the gauntlet, and then the roller to above the insertion of the deltoid, so as to secure an equable pressure; but he omitted to place any compress in the hollow of the hand. The limb was occasionally wetted through the bandage with marshmallow water.

Fifth day.—Fever has subsided. Countenance nearly natural; the inflammation of the arm and forearm diminished, but there were still darting pains in the hand, which remained livid and much swoln. The epidermis had peeled off, and a vesicle, about half an inch in size, had formed around the wound. The bandage was reapplied as before, *no compress being placed in the palm of the hand.* Brandy was added to the former lotion. The hand was placed upon a cushion; but severe pain and constant darting, with great heat, continued in it the whole day and next night.

Sixth day.—The arm and forearm better, but the back of the metacarpus was much tumefied, and appeared as if there was a deep-seated collection of matter. The wound was enlarged with a bistoury, but no pus escaped. The bandage was again applied, but in such a manner as to increase the pressure upon the fingers and the back of the hand; but there were still no compresses placed in the palm of the hand to ensure an equable compression.

Seventh day.—Arm and forearm well. The hand is less red, and not so much swoln, but remains very painful, and is described by the patient as feeling like the foot when a tight shoe has been worn. M. Velpeau was induced to believe that the greatest part of the suffering was owing to his want of precaution in not having filled the palm of the hand, in consequence of which the pressure was partially made. Having repaired this omission, the bandage was replaced rather looser than before, and during the day the pains ceased.

Ninth day.—Has passed a good night. Hand less swoln, but a gangrenous spot had formed at the extremity of each finger, and a part of the last phalanx of the forefinger was afterwards destroyed.

For some days the bandage was continued, and the patient was cured.

Case VI.—Gazeux, a servant, twenty-five years of age, of a sanguineous and robust constitution, was attacked, without any apparent cause, in September 1828, with pain, heat, and some swelling, around the left elbow. Fever succeeded, and M. Guerin de Manthelan was consulted, who, finding evident proof of gastric derangement, prescribed an emetic to be given the following day.

On the third day the fever was diminished, and the elbow less painful; but on the fourth and fifth day every symptom was much more severe.

M. Guerin was again called in on the sixth day. The whole of the forearm was now affected with severe darting pains, but still there was but little swelling. A large bread poultice was applied.

The patient was not again seen by the physician until the tenth day, at which time very severe phlegmonous erysipelas extended from the fingers to the upper part of the arm. The skin was red, and in some parts livid, particularly around the flexure of the elbow-joint, which was also extremely painful, hot, and dry; pulse 110, strong and full. Immediate recourse was had to compression. Although there was as yet no sensible swelling of the fingers, M. Guerin applied the gauntlet as a matter of precaution, and then a roller, as equably as possible, to within a short distance of the axilla, wetted with saturnine lotion.

Eleventh day.—Pain and swelling of the limb considerably diminished; but, the pulse still remaining hard and frequent, the patient was freely bled from the arm.

Twelfth day.—No fever. Has had some tranquil sleep. Inflammation now confined to the elbow, and even in that part is much diminished. There appears to be no longer any danger of suppuration.

For four or five days the bandage was continued, and the cure was completed in three weeks.

*Conclusions.* M. Velpeau considers it unnecessary to add to the number of detailed cases upon this subject. He has preferred the above instances, as they show that compression succeeds equally well in severe phlebitis which has existed for several days, (Case 1;) in phlebitis not less severe, but more superficial, and of more recent date, (2d and 3d Cases;) in inflammation of the lymphatic vessels complicated with erysipelas, and perhaps with phlebitis, (Case 4th;) in phlegmonous erysipelas of the severest kind, produced by wounds in dissection, (Case 5th,) whether it depends upon superficial or deep-seated phlebitis, upon injury of the lymphatic vessels, or merely upon inflammation of the subcutaneous cellular tissue, and in simple or phlegmonous erysipelas produced by any other cause, (Case 6th.) To confirm these various facts, M. Velpeau refers to cases he has before detailed in the Archives Générales, and to a case of phlebitis in the lower extremities in a pregnant woman, published by M. Goupil.* Six or eight equally conclusive cases have also been related by M. Guerin;† and M. V. has witnessed many other instances of ordinary erysipelas of the limbs, and after bleeding in the arm, cured by the above plan, but which were too similar to those described in 1826 to be again particularly mentioned upon the present occasion. His experience induces him to place such high confidence in the efficacy of pressure by means of bandages, that he does not hesitate to recommend it as "*un moyen héroïque*," as the most certain remedy which we possess against every kind of diffuse inflammation of the limbs, and other parts of the body which will admit of its exact application. In phlebitis, so long as the attack is limited to a small extent, and is slight in severity, we may perhaps rely upon leeches and poultices; but, as soon as the inflammation has a tendency to pass onwards towards the trunk of the body, or the surrounding cellular tissue

* Nouv. Biblioth. Med., Jan. 1827.

† Archives, Septembre 1827.

becomes inflamed, we should then have recourse to compression. If the disease does not extend beyond the commencement of the limb, and if abscesses are not formed either externally or internally, it is almost always arrested by this means; and even if the blood is contaminated by an admixture of pus, still pressure should be employed, because, by removing inflammation of the limb, the bandage destroys at least one source of mischief, and enables the constitution to resist other symptoms with more energy.

By incisions, punctures, or excoriations in dissection, both surgical students and their teachers not unfrequently lose their lives. If these injuries produce no general reaction, and remain strictly local, cauterization at first, and afterwards leeches and the usual topical applications, will in most cases be sufficient. If the wound is deep, and affects the tendons or their sheaths, or penetrates into a joint, the antiphlogistic treatment may be indispensable. But, whether the disease is transmitted by the veins, lymphatic vessels, or the cellular tissue, so soon as the inflammation extends to some distance beyond the wounded part, no other mode of treatment can at all be compared with compression. The septic nature of the disease does not prohibit it; for, provided it is possible to confine, as it were, the inflammation under the bandage, we shall rarely fail to limit its ravages, to diminish its severity, and even to dispel it altogether in the course of a few days.

Such is also the case in phlegmonous erysipelas, or diffuse phlegmon; and M. Velpeau expresses his surprise that in a disease which practitioners confess is one of the fatal shoals of surgery, we should still persist in the application of leeches and emollients, or in making deep and numerous incisions upon the inflamed parts, instead of having recourse to a simple compressing bandage. No doubt, general bleedings, of fifteen, twenty, or thirty ounces, for three or four successive days, according to the practice in England, or the numerous incisions recommended by A. C. Hutchison, Beauchêne, Bodson, Vincent, &c., when the disease is in an advanced stage, is to a certain extent successful, as is proved by the practice at the hospital of Saint-Antoine, and the memoir by Mr. Lawrence,* but such a mode of treatment cannot fail to have a prejudicial effect upon the general health of the patient, leaving out of the question the suffering it inflicts. Compression disturbs none of the functions of the body, and cures much

* Med. Chir. Trans. vol. xiv. 1828.

more frequently and more promptly than all the bleedings and incisions that can be imagined. It follows, then, according to M. Velpeau,

1st. That, to prevent phlebitis after amputations, it is prudent, as is constantly practised by M. Richerand at the hospital Saint Louis, to establish an exact but moderate compression from the commencement of the limb to within a short distance of the wound; which we should, besides, endeavour to unite as soon as possible.

2d. That, by the aid of a *well-applied* circular bandage, phlebitis will seldom occur, and very rarely be dangerous, after those operations which are practised by some surgeons for varicose veins of the legs.

3d. That phlebitis, erysipelas from dissection wounds, or ordinary erysipelas, may be almost constantly arrested and cured by dexterously applied compression, if it is had recourse to before suppuration is completely established; and that, in all these diseases, compression ought to constitute the chief remedy; while bleeding, leeches, incisions, &c. are only to be occasionally employed as auxiliaries.

M. Velpeau has before* mentioned the precautions which the application of the bandage requires, and described its mode of action. He states it to be a mechanical agent, the efficacy of which entirely depends upon the hand that applies it; and that, if it fail in cases which appear to indicate its employment, the surgeon, and not the remedy, is mostly in fault. If the pressure be not regular, or either too great or too little in any point; if the various hollows and inequalities of the limb be not adroitly made level with the surrounding surface, then, in all probability, the pain will be increased instead of diminished, and other perplexing symptoms be produced. As a proof of the strict attention required to ensure an equal pressure from the bandage upon *every part*, the fifth case is referred to, in which, confessedly, proper precautions were not at first taken, and consequently bad symptoms arose, and relief was delayed. The pressure should commence as far as possible below the inflamed part, and be carried considerably above it.

M. Velpeau is not acquainted with any author who has expressly said that compression by means of a bandage was the best mode of treating phlebitis, or phlegmonous erysipelas without solution of continuity, or those acute inflammations which result from wounds in dissecting. In the

* Archives, Juillet 1826.

sixteenth volume of the Dictionnaire de Médecine, there is, indeed, this expression: "Slight methodical compression made upon the limb has frequently succeeded," as is proved by a case related by M. Goupil, in the Bibliothèque Med. Janvier 1827; but M. Velpeau had previously stated, in commenting upon phlebitis,* "that, whether the tumefaction of the arm which results from a lancet wound depends upon inflammation of a vein or the subcutaneous cellular tissue, does not at all affect the question of bandaging the part. In each case compression offers the same advantages." In different works there are certainly facts related which ought to have led to this practice. The case reported by A. Paré describing the effects arising from bleeding the king of France, which had well-nigh proved fatal, must be considered as one of phlegmonous erysipelas, if not of phlebitis; and the danger was certainly much more averted by a compressing bandage than by any of the other remedies employed. When Hunter proposed to compress the vein between the trunk and the injured or diseased part, for the purpose of limiting the inflammation, would it not, M. Velpeau asks, have been more simple and natural to have applied pressure over the whole extent of the limb? The success obtained from this practice by M. Bretonneau in burns, and contused wounds complicated with gangrenous erysipelas, was sufficient to lead to the inference that it would be equally useful in diffuse phlegmon, or in the inflammations arising from wounds with instruments soiled by deleterious matter.

In conclusion, M. Velpeau remarks that the facts hitherto published by himself or others upon this question may not be sufficiently numerous or conclusive to establish that conviction in the minds of the profession in general which he himself feels. He is aware that he may be accused, perhaps, of over-estimating the importance of the practice he so warmly recommends. His wish is not to establish axioms, but merely to induce other surgeons to judge for themselves, and to repeat his experiments with all the care, and precisely in the manner that he has described; and he feels confident that the result will be much more favorable than may at present be anticipated.

---

The efficacy of compression in different kinds of diffuse inflammation of the extremities, does not rest upon the authority of M. Velpeau alone. In 1815, Bretonneau

* Archives, tom. ii. p. 405.

defended a thesis, which was indeed very indifferently received by the professors, the object of which was to show the utility of compression in phlegmonous erysipelas. In the same year, also, Dr. Balfour, of Edinburgh, recommended the same treatment in chronic and even acute rheumatism;* a disease which, although differing in many respects from erysipelas and the other inflammatory affections for which compression by bandages is advised by the French physicians, is by no means so essentially different from them as to render it improbable that in each the same treatment might apply. Dr. Balfour supported his doctrine with ingenuity, and detailed many cases which appeared satisfactorily to prove the benefit of applying moderately tight bandages in cases of rheumatism, however much the local suffering and appearances seemed to contra-indicate such a mode of treatment. The practice, however, has never been very generally adopted.

Many severe cases of diffuse inflammation, arising from various causes, have recently been published in the continental journals, in which compression was the chief and very beneficial remedy. But although, with such strong additional evidence before us as that which has been adduced by M. Velpeau, we cannot doubt the high merit of the treatment by compression, we conceive he places too implicit a reliance upon it. He sometimes either altogether omits, or at least very ineffectually resorts to, constitutional treatment.

To encourage other surgeons to adopt the practice, M. Velpeau informs us that he has never seen any ill effects arise from its *judicious* employment, during ten years' extensive trial of it. Without this assurance, some apprehension might be felt by those who have not had personal experience of the safety of compression, of applying it in cases of severe local inflammation, where it might be imagined the slightest pressure could not be borne. For some time after the application of the bandage, the pain, it appears, is much increased; but we are not to remove it on this account, as the suffering afterwards gradually diminishes, together with every other symptom.

To communicate an apparently very valuable practical fact, has been our only object. Those who are desirous of knowing the theory of the *modus agendi* of the treatment so strongly recommended, may consult Dr. Balfour's paper,

* Observations on the Pathology and Cure of Rheumatism; by WILLIAM BALFOUR, M.D.—Edinburgh Med. and Surg. Journal, vol. xi p. 168.

to which we have already referred, and the memoir of M. Velpeau, in the Archives Générales, Juillet 1826, p. 425. In the Traité de Médecine Opératoire of M. Roux, and that of Sabatier, and the Elemens de Pathologie de MM. Roche et Sanson, much interesting information is contained upon the general subject of compression as a remedial agent in disease.—Editors.

